# EVtracker: An Event-Driven Spatiotemporal Method for Dynamic Object Tracking

**DOI:** 10.3390/s22166090

**Published:** 2022-08-15

**Authors:** Shixiong Zhang, Wenmin Wang, Honglei Li, Shenyong Zhang

**Affiliations:** School of Computer Science and Engineering, Macau University of Science and Technology, Avenida Wai Long, Taipa, Macau

**Keywords:** event-based camera, object tracking, spatiotemporal method

## Abstract

An event camera is a novel bio-inspired sensor that effectively compensates for the shortcomings of current frame cameras, which include high latency, low dynamic range, motion blur, etc. Rather than capturing images at a fixed frame rate, an event camera produces an asynchronous signal by measuring the brightness change of each pixel. Consequently, an appropriate algorithm framework that can handle the unique data types of event-based vision is required. In this paper, we propose a dynamic object tracking framework using an event camera to achieve long-term stable tracking of event objects. One of the key novel features of our approach is to adopt an adaptive strategy that adjusts the spatiotemporal domain of event data. To achieve this, we reconstruct event images from high-speed asynchronous streaming data via online learning. Additionally, we apply the Siamese network to extract features from event data. In contrast to earlier models that only extract hand-crafted features, our method provides powerful feature description and a more flexible reconstruction strategy for event data. We assess our algorithm in three challenging scenarios: 6-DoF (six degrees of freedom), translation, and rotation. Unlike fixed cameras in traditional object tracking tasks, all three tracking scenarios involve the simultaneous violent rotation and shaking of both the camera and objects. Results from extensive experiments suggest that our proposed approach achieves superior accuracy and robustness compared to other state-of-the-art methods. Without reducing time efficiency, our novel method exhibits a 30% increase in accuracy over other recent models. Furthermore, results indicate that event cameras are capable of robust object tracking, which is a task that conventional cameras cannot adequately perform, especially for super-fast motion tracking and challenging lighting situations.

## 1. Introduction

Event cameras have attracted more and more attention from researchers due to their excellent capturing performance for moving targets [1,2,3,4]. An event-based camera, also known as a neuromorphic camera or dynamic vision sensor (DVS), is a new type of sensor closer to biological vision than conventional frame-based cameras. Therefore, it has advantages such as low power consumption (1 mW), high dynamic range (140 db), extremely high temporal resolution, and low latency (microsecond level) [5,6].

**Figure 1:**
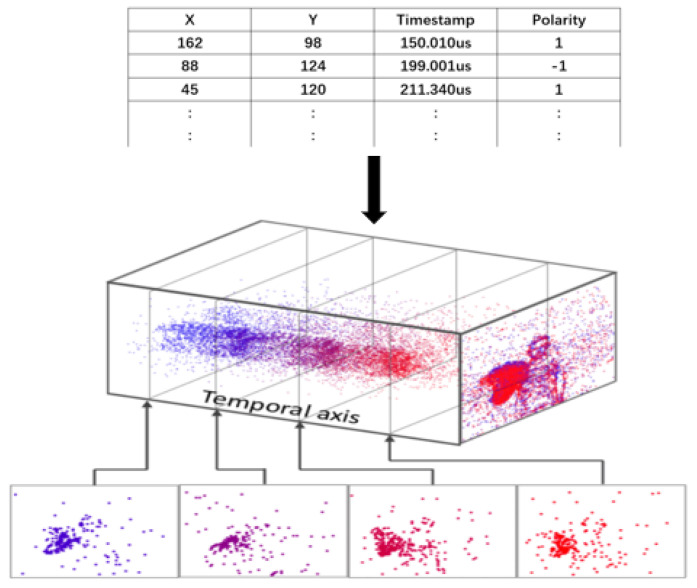
This picture shows how the event camera captures event data. When a new event is triggered, the event camera will only update the coordinates of the activation point instead of the full image. Event data generally include four parts: timestamp, coordinate information (x, y), and polarity. We can observe from the figure that each event data point is asynchronous. Event data is dense in the temporal domain and sparse in the spatial domain. (Data source adapted with permission from [10]. December 2020, Elsevier).

These capabilities enable event cameras to be widely used in autonomous driving and intelligent transportation, drones, and so on [7,8,9]. Nevertheless, compared with the large number of mature applications of conventional cameras, the related algorithms and applications of event cameras are still very lacking. If we want event cameras to play a significant role in real systems, we still have significant work to do. Fortunately, some computer vision algorithms can be improved and applied to event cameras, especially some algorithms based on video sequences, such as object tracking, optical flow, etc. As shown in Figure 1, each event pixel (blue dot) captured by an event camera with a different timestamp is distributed in a spatiotemporal domain; conventional computer vision algorithms cannot handle such discrete event data. This requires the reconstruction of event data into frames that are similar to traditional image frames. The strategy of reconstructing event data can bridge the gap between conventional computer vision and event-based vision. Thus, a convolutional neural network can be effectively used to extract features from the reconstructed event images.

In this paper, we proposed an event-driven spatiotemporal domain adaptation method for dynamic object tracking. We name our proposed event-driven tracking framework EVtracker.

Object tracking is a hot topic in the field of computer vision. Loosely speaking, given the initial position of an object in the video, an object tracking algorithm can track the constantly moving object in the video sequence. Compared with object detection and object classification, object tracking pays more attention to the object’s trajectory in a time series. Research on visual object trackers is a very active field. Each year, new object tracking algorithms are proposed with demonstrated successes [11,12,13,14]. However, there are still some challenging scenes that restrict the improvement of the visual tracking algorithm, such as background clutters and motion blur [15,16]. Event cameras provide new ideas for solving these challenges of visual tracking tasks [1,17,18]. In other words, the event object tracking algorithm can provide a low-latency and wide-dynamic tracker. We propose dynamic object tracking for event vision that integrates traditional visual tracking algorithms and event cameras.

This paper introduces a long-term tracking method based on event cameras. We propose a simple and effective method to balance the spatial and temporal domains of event cameras. Due to the high update frequency of the event camera, the event camera has a spatial sparsity and higher temporal resolution. Therefore, the event data is dense in the temporal domain and sparse in the spatial domain, as shown in Figure 1. Our method is to reconstruct the event data in the spatial domain by evaluating the quality of the features of the object. Following the works of e-TLD [19] and e-LOT [20], we propose a novel approach to reconstructing event data and tracking event objects. Compared with e-TLD and e-LOT, our dynamically reconstructed event images are clearer with higher quality features, and we design and train more powerful deep networks instead of using their hand-crafted features. The main contributions of our paper are summarized as follows:

- We developed a straightforward and effective tracking and detection framework for long-term tracking from an event camera. We demonstrated in detail the many advantages of using event cameras for object tracking;
- We propose a novel approach to dynamically reconstructing event images for object tracking by evaluating feature quality. Experiments show that our method can effectively adjust event data in the spatiotemporal domain under different scenarios;
- Our method can effectively balance spatial resolution and temporal resolution in the tracking field; the experiments show that our proposed approach has better performance than the existing state-of-the-art event-based tracker and frame-based tracker.

We have organized the rest of this article in the following way. Section 2 describes the related work on event-based vision and visual object tracking. In this part, we review related research and articles. Section 3 introduces our EVtracker and presents the details of our proposed method for event-based vision. Section 4 shows our experiments and ablations, and we discuss our proposed method. The paper ends with conclusions, and we also present our future research in Section 5.

## 2. Related Work

In this section, we will first introduce event-based vision and its latest applications, then introduce the related research on visual object tracking. Finally, we will review several methods for tracking using event cameras.

### 2.1. Event-Based Vision

An event camera, also known as a dynamic vision sensor (DVS), is a sensor that responds to local changes in brightness. Event cameras do not use synchronized shutters to capture images like traditional frame cameras. Instead, each pixel within the event camera operates independently and asynchronously, only activating when a change in brightness occurs. Event cameras can effectively supplement the existing deficiencies of computer vision and robot vision. In the survey paper [5], Guillermo Gallego et al. provided a detailed and comprehensive description of the emerging field of event-based vision. They summarized the working principles and the latest development in event cameras. This review shows that researchers are using this revolutionary type of camera to reformulate some tasks in computer vision, such as SLAM, object tracking, motion capture, optical flow, etc. [21,22,23,24,25].

Research and applications related to event-based vision are constantly increasing [6,8,26,27]. Antoni et al. [28] propose an ultimate SLAM that integrates multiple sensors, such as event cameras, in high-speed scenes. EventCap [29] proposes an approach to capture high-speed human motions using a single event camera; this system can capture high-speed human poses with low data bandwidth and power consumption. In the paper [30], the authors provide an exciting algorithm that uses event cameras for star tracking. Since the original event pixels are discrete and asynchronous, most of these algorithms use a parameter to generate event images by gathering a fixed time window or a fixed number of event pixels. This is very conducive to processing event camera data; however, fixed parameters cannot adapt to changes in multiple scenes and the parameter values rely on human experience and observations. These articles do not effectively balance the spatial and temporal distribution of event data. Our paper provides a new framework for object tracking based on event cameras that can dynamically adjust the spatiotemporal domain to synthesize event images according to different scenes.

### 2.2. Visual Object Tracking

In the past decade, there have been various typical models of visual tracking. The L1 trackers [31,32] and compressive trackers [33,34] proposed sparse matrix operations for tracking. TLD [35] combined the tracking model and detection model for long-term tracking. SPT [36,37,38] presented the tracking algorithm within a discriminative tracking approach based on a super-pixel representation of the videos. Despite all of these trackers demonstrating success with hand-crafted features in past years, the performances still can be improved by using powerful feature descriptions.

In the last few years, convolutional neural networks (CNNs) have significantly improved the performance of visual object tracking [13,39,40]. Siamese network-based trackers are constantly being proposed and improved, such as SiamFC [41], SiamRPN [42], SiamMask [43], and SiamRPN++ [44]. These algorithms have attracted much attention because of their excellent performance in various benchmarks. Siamese network-based trackers formulate the tracking task as a patch matching problem; they provides two branch networks with shared weights to establish target templates and search for candidate targets, respectively. Their performance is still expected to be improved under motion blur, illumination, and cluttered background. A high frame rate camera may be able to overcome the motion blur problem, but at the same time, it brings the challenges of high power consumption and high data redundancy. Moreover, high frame rate cameras are sensitive to illumination. Our proposed approach provides a novel solution to the inherent object tracking challenge. We hope that our approach can inspire more object tracking work with event cameras.

### 2.3. Event Camera Based Tracking

Object tracking based on event cameras demonstrates the potential to solve tracking challenges. Tracking algorithms based on event cameras are constantly being proposed to be applied in different scenarios. Due to the good perception ability of the event camera for moving scenes and complex lighting scenes, it is often used in challenging object-tracking tasks. For instance, Afshar et al. [45] and Chin et al. [46] propose the use of event cameras for object tracking in space scenes. Some other works focus on the use of event cameras for object tracking in traffic scenes, e.g., autonomous driving, drones, autonomous robots, etc. [47,48,49,50]. Cao et al. [51] propose an event object tracking method using a spiking neural network and apply it in an autonomous robotic system. Although the spiking neural network adapts well to asynchronous event streams, its performance is still limited. Li et al. [52] propose an event-based object tracking method based on deep features and correlation filtering. Liu et al. [53] and Wang et al. [54] adopt the strategy of fusing event data and frames for the object tracking task. These methods simply convert the event data into a fixed matrix and do not take advantage of the asynchrony and high temporal resolution of the event camera.

Both e-TLD [19] and e-LOT [20] are very related to our proposed approach. e-LOT proposes a long-term object tracking and detection framework for event cameras and a distribution aware retinal transform as a generic visual descriptor of event data. This feature descriptor is based on the log-polar. However, this descriptor can only be based on a clean background, relies on hand design rather than being data-driven, and performs poorly in complex scenes. e-TLD effectively extends the method of e-LOT and proposes a data-driven long-term tracking framework, including a local sliding window-based detector. It uses the detector to re-initialize the tracker after a failed track. To the best of our knowledge, these methods have the following limitations: the description of the event data of these methods is not good enough, the event image with a clear spatial structure cannot be reconstructed well, the feature descriptions extracted by these methods are not powerful enough and rely too much on hand-crafted features, and these methods do not adopt the latest visual tracking algorithms for the event camera, relying on some outdated algorithms [35].

Generally speaking, there are far fewer event-based camera tracking methods than traditional visual tracking. One of the main challenges is that the asynchronous event streams cannot be dealt with by current convolutional networks, which are being designed based on synchronous frames. In our paper, we use the spatiotemporal domain adaptation method to reconstruct event images with clear spatial structure. We also provide learned features with strong characterization capabilities to describe the object based on event cameras.

## 3. Methodology

In this section, we will first introduce the motivation of our proposed method. We will describe in detail our strategy for adjusting event data in the spatiotemporal domain. After that, we will present our tracking algorithm: EVtracker for event cameras.

### 3.1. Motivation

A visual object tracker provides a way to track objects in video sequences. Compared to other computer vision tasks such as object detection and image segmentation, object tracking algorithms extract not only spatial features, but also features in the time series. However, frame-based trackers only focus on spatial resolution and ignore temporal resolution, which is due to the principle limitation of conventional cameras. Event cameras show their unique potential in the object tracking task due to their high temporal resolution and asynchronous data. Event cameras provide a novel solution to the inherent challenges of object tracking. To better enable an event camera to serve the object tracking task, we propose our approach to balancing the temporal and spatial distribution of event cameras.

In Table 1, we summarize the features of event cameras and frame cameras. Due to the large difference between event and frame cameras in principle, their different characteristics are reflected in the spatiotemporal domain, dynamic range, and update model. Event cameras provide highly dynamic, high temporal resolution data at lower power and data volume, with more detailed advantages [5].

In this paper, we provide a novel method that can dynamically adapt to different challenges of object tracking tasks. Conventional cameras cannot adjust spatiotemporal information according to the various scenes, while event cameras can do this well. Our method uses these advantages to overcome tracking challenges such as motion blur, fast motion, illumination variation, etc.

A feature of an event camera is that it can capture each event pixel asynchronously. Due to the high update frequency and low latency of an event camera, an event camera has a higher temporal resolution. On the other hand, an event-based camera only captures the variation in log-scale intensity, so the event data is sparse in the spatial domain. However, existing visual algorithms are good at processing spatial information. Most event camera-based algorithms are processed by reconstructing event images, such as human pose recognition based on event cameras [55] and object tracking based on event cameras [52,53,54]. These methods adjust the spatiotemporal neighborhood by counting a certain number of events or accumulating polarity and generating a two-dimensional frame/image compatible with conventional visual computer vision algorithms [5]. However, they ignore that the flow of events constantly changes in different scenarios and the feedback of conventional visual algorithms to event features. Thus, we would like to ask a research question: Is it possible to dynamically adjust the two-dimensional description of event images according to different scenes? Therefore, reconstructing event images in the spatial domain is a meaningful exploration of event vision. In response to this research question, we propose a strategy for dynamically reconstructing event images in Section 3.2, and adapt this method to long-term object tracking tasks in Section 3.3.

### 3.2. Spatiotemporal Domain Adaptation

In this part, we present the spatiotemporal domain adaptation method for event-based object tracking. Our strategy is very straightforward and effective. If this two-dimensional event image can provide high-quality features, it can be judged that this event image has a clear two-dimensional event image. The quality of features can be evaluated through a shallow network. Generally, an event pixel *e* is represented as a 4-tuple (x,y,ti,p), where *x* and *y* denote the spatial coordinates of an event pixel, *p* represents the event’s intensity, and ti is a timestamp. The sensor of the event camera independently measures the change of the intensity $I_{ti}$ of each pixel and provides an asynchronous event flow with microsecond resolution. When the variation in logarithmic brightness exceeds the threshold *C*, ∣▽L∣≥C, we formulate this process as follows:

(1) $$|L\left(I_{ti+▽ti}\right)-L\left(I_{ti}\right)|\geq C,$$

where ▽ti is the time interval at the microsecond level. *L* is the log-scale of intensity *I*: $L\left(I_{ti}\right)=log\left(I_{ti}\right)$; when the light brightness increases or decreases, the event pixels will be activated with an event polarity p∈{−1,1}, where 1 and −1 represent the increase and decrease of brightness, respectively.

From the above introduction, we can know that the event pixel is an independent asynchronous update. However, only an image patch of event data has spatial features; we define a spatial event frame P(h×w) to gather event pixels to form an event image, where *h* and *w* are the height and width of the event sensor, respectively. In the existing methods, one of the strategies for reconstructing event images is to collect the polarity of event pixels one by one over time interval ▽T on the entire image patch, where ▽T is a specific time interval for time windows $\left[T_{start},T_{end}\right]$; apart from this, another strategy is to use a fixed number of event pixels *N* for an image patch. Although both strategies have been proven successful in some applications through careful manual design, there are still concerns about their reliability and robustness.

Therefore, we propose a novel strategy in which adaptive adjustments can be made to ▽T and *N* according to the different scenes. There are two ways to represent each spatial event frame: time-window $P_{▽T}$ and fixed number of events $P_{N}$. We map the polarity of the event pixel to the image pixel range [0:255]. Our strategy is divided into two main parts: initialization of online learning and dynamic adaptation in time series.

#### 3.2.1. Initialization of Online Learning

Unlike other methods that use experience and hand-craft to define fixed ▽T and *N* [28,29,54], we first search for the initial ▽T by online learning. An event frame representation is computed as:

(2) $$P_{▽T}=\int_{T_{start}}^{T_{end}}e\left(t\right)dt,$$

where *P* is the aggregation of all asynchronous event pixels in the time windows $\left[T_{start},T_{end}\right]$. The optimal event frame $\hat{P}$ is computed by the heatmap estimation

(3) $$\hat{P}=\underset{▽t\in [0:n]}{\arg\max}\left(\;f\left(P_{▽t}\right)\right),$$

where *f* represents the response of the feature, $P_{▽t}$ represents the candidate description, *n* is the number of samplings in a time series, and higher heatmap response means a higher quality of the event image. Features are extracted and evaluated by a shallow trained network *f*. The shallow network structure includes convolutional layers and CSPblocks(Cross-Stage Partial Connection Blocks); we borrowed CSPblocks from the YOLOv4-tiny model [56]. After this shallow network is pre-trained with event data, this network can effectively extract event image features, similar to traditional vision tasks. We use online learning strategies to generate the initial ▽T, which evaluates the time window of continuous accumulation ▽T until we find an optimal time window $P_{▽T}^{1}={\hat{P}}_{▽t}$. When we obtain an optimal time window, it means we obtain a starting frame for object tracking. We acquire an initial time interval ▽T for event image reconstruction, which is essential for our subsequent strategy. At the same time, we can count the number of event pixels *N* contained in this time window.

#### 3.2.2. Dynamic Adaptation in Time Series

**Figure 2:**
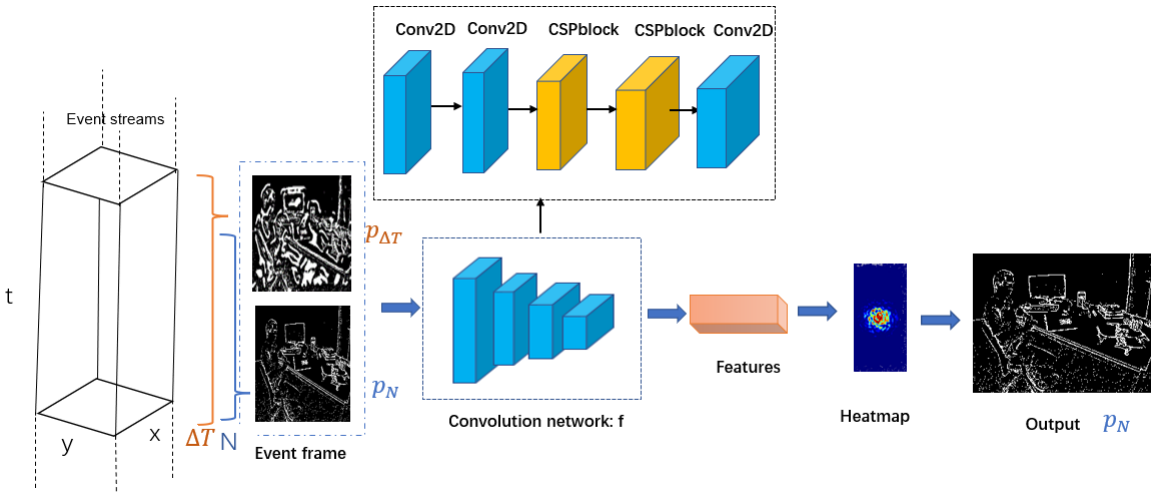
Overview of our reconstruction strategy of the event image, Event streams represent an event flow of asynchronous data that is updated on a time series. The event frame indicates the candidate event image. The CNN indicates a shallow convolutional neural network to extract features. By comparing features, a higher heatmap means that the quality of the features is better and a better event image can be output. In this case, $P_{N}$ provides higher-quality event images with better texture features.

The object tracking algorithm is intended to track a specific target in a time series, and the event image sequence needs to be continuously generated. We adopt a dynamic adaptive strategy to generate new event images continuously. Our strategy is effective based on the characteristics of the event camera. The movement speed of the object or camera will affect the distribution of the event data in the space–time domain. In the same event interval ▽T, different movement speeds will produce different numbers of event pixels *N*. Therefore, no matter whether a fixed time interval or a fixed number of event pixels is used to reconstruct the event image, it cannot adapt to the changes in the scene. We assume that the changes in the time series are progressive and continuous; when new event data comes, we apply the parameters obtained in the previous stage to reconstruct the event image and we obtain $P_{▽T}^{2}$ and $P_{N}^{2}$. Then, we use Equation (3) to evaluate them and compare the evaluation results.

(4) $$v=\mathrm{compare}({\hat{P}}_{▽T}^{2},{\hat{P}}_{N}^{2}),$$

where *v* is the greater of ${\hat{P}}_{▽T}^{2}$ and ${\hat{P}}_{N}^{2}$. If ${\hat{P}}_{▽T}^{2}$ is optimal, we recount *N*, and if ${\hat{P}}_{N}^{2}$ is optimal, we update ▽T, where ${\hat{P}}_{▽T}^{2}$ and ${\hat{P}}_{N}^{2}$ are the heatmap response of $P_{▽T}^{2}$ and $P_{N}^{2}$, respectively. We use an alternate update strategy to continuously update ▽T and *N* in the time series. We use dynamic time intervals and the number of event pixels to balance the event data in the spatiotemporal domain. An overview of the reconstruction strategy is shown in Figure 2. We convert the three-dimensional asynchronous event stream into a two-dimensional event image, and our method can generate event images with better texture features regardless of scene changes. It is important to note that our adaptive adjustment can adjust the high-speed asynchronous event stream, which is impossible with traditional cameras.

### 3.3. EVtracker

**Figure 3:**
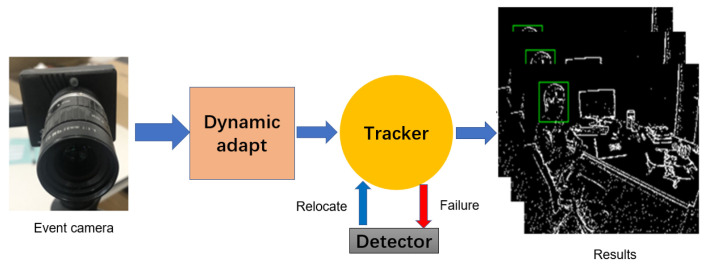
This picture shows the real system using our proposed framework, event camera output event flows, and a dynamic adaptive strategy to reconstruct the event stream into an event image in a time series. Our tracking algorithm tracks the object in the event image sequence. When the tracking fails, the detector is used to reinitialize the tracker.

In this part, we mainly introduce our tracking strategy; we adopt the Siamese network as the baseline of our event-based tracking algorithm. To track an event-based object long-term, we follow the strategy of the articles [19,57] by setting up a detector to relocate the target when the tracking fails, as shown in Figure 3.

#### 3.3.1. Siamese Network Tracker

**Figure 4:**
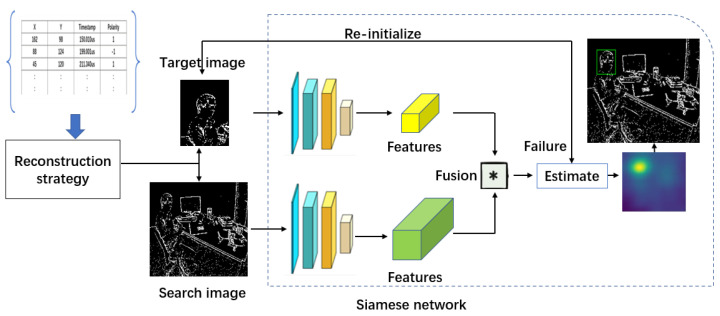
This picture shows an event camera-based tracker using the Siamese network, with feature extraction of the target image and search image using a shared weight network.

The Siamese network tracker is a baseline for many recent popular algorithms [58]. We propose a tracking framework based on a Siamese network, as shown in Figure 4. These networks can implicitly encode the original event patch into feature space and then use a specific tensor to fuse them to produce a single output. They usually compare the features of two branches in the implicit embedding space. Similar to video sequence tracking, we regard the learning of tracking targets in event vision as the learning of similarity problems. One of the branches is called the template branch and we define its output features as $\varphi (p_{y})$; it receives the target patch in the historical frame as input. The other is called the search branch and its output feature is defined as $\varphi (p_{z})$; it receives the image patch in the current frame as input. The object tracking of an event camera is formulated by the classification module and the regression module:

(5) $$\begin{array}{cc} \; & H_{h\times w\times 2k}^{cls}={\left[\varphi \left(p_{y}\right)\right]}_{cls}⊗{\left[\varphi \left(p_{z}\right)\right]}_{cls},\\ \; & H_{h\times w\times 4k}^{reg}={\left[\varphi \left(p_{y}\right)\right]}_{reg}⊗{\left[\varphi \left(p_{z}\right)\right]}_{reg}, \end{array}$$

where $H_{h\times w\times 2k}^{cls}$ and $H_{h\times w\times 4k}^{reg}$ denote classification map and regression map, respectively, ⊗ represents the convolution operation, and 2k and 4k represents the number of channel vectors they contain; these vectors measure the distance between the candidate anchor and the ground truth. $p_{y}$ and $p_{z}$ are the input event images of the template branch and the search branch, respectively.

The classification function needs to distinguish the foreground and background of the tracking object. Here, we provide a classification loss function as:

(6) $$L_{cls}=-\sum_{i=0}^{1}C_{i}log\left(S_{i}\right),$$

where $C_{i}$ is the label of the classification and $S_{i}$ is the probability of the correct classification. In the regression module we provide $\left(x_{c},y_{c},w_{c},h_{c}\right)$ to represent the tracking prediction box and $\left(x_{g},y_{g},w_{g},h_{g}\right)$ represents the ground truth. The distance is normalized as:

(7) $$\begin{array}{ccc} \; & \delta \left[0\right]=\frac{x_{g}-x_{c}}{w_{c}}, & \delta \left[1\right]=\frac{y_{g}-y_{c}}{h_{c}},\\ \; & \delta \left[2\right]=ln\frac{w_{g}}{w_{c}}, & \delta \left[3\right]=ln\frac{h_{g}}{h_{c}},\;\;\;\; \end{array}$$

The regression loss is provided as follows:

(8) $$l_{reg}=\sum_{i=0}^{3}smooth_{L_{1}}\left(\delta \left[i\right]\right),$$

Then, the smooth $L_{1}$ loss can be written as follows:

(9) $$smooth_{L_{1}}\left(x\right)=\left\{\begin{array}{ccc} & 0.5x^{2},\;\;\;\;\;\; & if\left|x\right|<1,\\ \; & \left|x\right|-0.5, & otherwise. \end{array}\right.$$

The total loss function in object tracking can be expressed as:

(10) $$L_{loss}=L_{cls}+\lambda L_{reg}$$

where λ is a hyperparameter to balance classification loss and regression loss.

#### 3.3.2. Re-Initialize Tracker

Our dynamic reconstruction strategy can solve the problem of fast motion and target drift in object tracking well, though long-term tracking remains a challenge, as the target may leave the field of view for a long duration. To be able to track the object in event-based vision long term, we set up a detector *D* to update the target template. The detector can perform a global search of the event image without requiring the previous position of the target, so it can reinitialize the tracker when the tracking fails. However, judging tracking failure is a challenging problem. One of the common strategies is to use a threshold θ to estimate whether the tracking is successful, i.e., if the tracking fails, then the tracking score is less than this threshold. Initially, we use the detector to extract features from the first-frame ground truth bounding box. Afterward, we re-run the detector on the current frame and compute the similarity score between each pair of detections. The higher the similarity, the higher the probability that they are the same object, and the latest position information of the target is updated in the tracking model. The similarity score is obtained by evaluating the distance of the detector features. Therefore, when the tracking fails, we run our detector for global detection; then we obtain candidate objects $\left(D_{1},D_{2},D_{3},....,D_{j}\right)$ and evaluate the similarity of these candidates to the original tracking target template; we have

(11) $$p_{j}=\underset{j\in [1:m]}{\arg\max}\left(\varepsilon_{j}\left(sim\left(D_{j},p_{z}\right)\right)\right),$$

where $D_{j}$ represents the candidate target for detection, $p_{z}$ represents the tracking target template, sim(·) is calculating cosine similarity of features, and $p_{j}$ represents the re-initialization target. To be able to suppress distractors, we set the weight parameter $\varepsilon_{j}$, which is obtained by calculating the center location Euclidean distance between the current frame detection candidate and the tracking position of the previous frame, so we have $\varepsilon_{j}=\frac{1}{Eu(D_{j},P_{t-1})}$, where Eu(·,·) represents the Euclidean distance for calculating the center position. The smaller the distance, the larger the weight parameter $\varepsilon_{j}$. Inspired by the article [57], we use a combination of parameter judgment and regular updates to solve the relocation problem after tracking drift. We provide a novel framework that integrates tracking and detection to learn and solve long-term tracking problems.

## 4. Experiments

**Figure 5:**
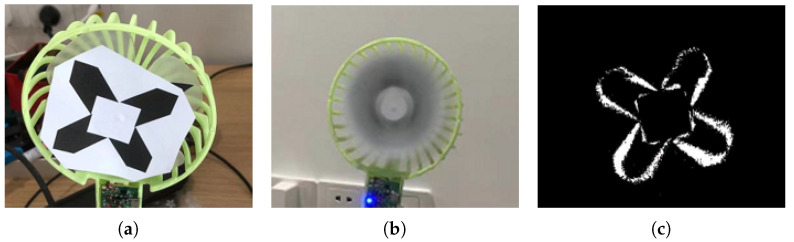
(**a**) shows the original image when the fan is stationary, (**b**) shows the image taken with an RGB camera; when the fan rotates at high speed, the picture can no longer be observed due to motion blur. (**c**) shows the event data taken with an event camera when the fan rotates at high speed; we visualize event data for comparison and a clear target structure without background is captured. It can be observed from this comparison diagram that the event camera can effectively avoid motion blur.

In this section, we provide four experiments to evaluate our method. First, to better explain the advantages of the event camera, we designed an experiment to verify the advantage of event cameras in capturing fast-moving objects, as shown in Figure 5. Then, we compare our method with recent state-of-the-art event-based tracking methods [19,20] and state-of-the-art frame-based tracking algorithms [42,43,44] on a series of challenging datasets. In addition, we designed an ablation study to analyze the effectiveness of our strategy. We will make our code and demos available at: https://github.com/yexuezhaoge/EVtracker, https://www.youtube.com/watch?v=0_PfpfxkCTQ (accessed on 11 June 2022).

Success Plot and Precision Plot are currently popular tracking algorithm evaluation metrics. We use these two evaluation metrics to evaluate the performance of our proposed approach. All of the evaluation has been presented in benchmark paper [8,15]. The first evaluation metric, Success Plot, indicates the percentage of frames when the overlap between the prediction box of the tracking algorithm and the ground truth is higher than a set value. The overlap is defined as $overlap=\frac{area(R_{t}⋂R_{g})}{area(R_{t}⋃R_{g})}$, where $R_{t}$ is a tracked bounding box and $R_{g}$ is the bounding box provided by the ground truth. We set the overlap values at a threshold of 0.5; the success plot is defined as the relative number of frames in the video sequence when the overlap>0.5.

The second evaluation metric, Precision Plot, demonstrates the percentage of frames where the CLE between the tracked object position and the ground truth is smaller than a pre-set threshold. CLE is computed as the Euclidean distance between the center locations of the target and the manually labeled ground truth, as follows:

(12) $$CLE=\sqrt{{\left(x_{g}-x_{i}\right)}^{2}+{\left(y_{g}-y_{i}\right)}^{2}}$$

where $\left(x_{g},y_{g}\right)$ and $\left(x_{i},y_{i}\right)$ are, respectively, the *x*, *y* coordinates of the center locations of the ground truth and the detected object in the *i*-th frame.

**Implementation details:** All the experiments with the proposed EVtracker were executed on a server with an Intel Xeon(R) Silver 4114 CPU@2.20GHz × 40.

**Figure 6:**
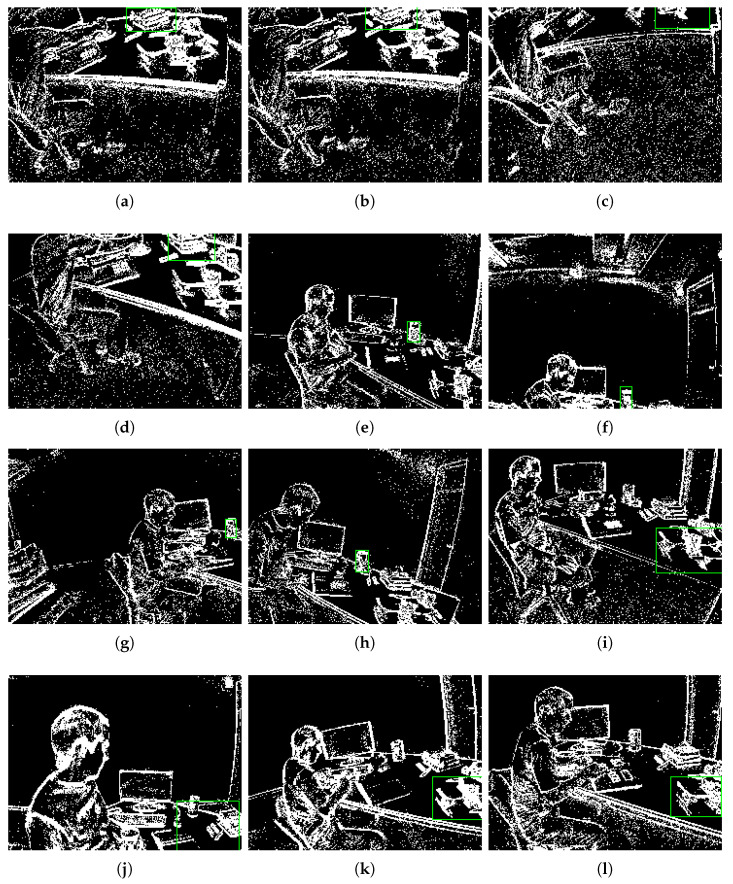
Tracking objects of different shapes and sizes. Each row shows the tracking of a single object; (**a**–**d**) show the tracking results of the book; (**e**–**h**) show the tracking results of the cup; (**i**–**l**) show the tracking results of the drone. It is shown here that our method can overcome the challenges of dramatic scene changes, object out of view, etc., and has good robustness.

In our EVtracker, we adopt SiamRPN [42] as the baseline of our Siamese network; we utilize the pre-trained AlexNet [59] for feature extraction in the tracking model. In particular, detector *d* and feature extractor *f* share the same backbone, and features of event data only need to be extracted once. Due to the lack of large-scale event datasets, we annotated 3000 event data for model pre-training. We define threshold θ=0.5 to determine whether the tracking fails. We set the threshold of CLE to the default value of 20 pixels. Because we are using shallow neural networks, the original input size of the event image is 240 × 180, and our method does not require many computing resources. Feature evaluation and object detection can share a shallow network and our proposed EVtracker can be run in real time with a speed of 67 FPS. According to our experience, we set *N* = 30,000 and ▽T=30 m in our ablation study. Figure 6 shows some visual results of our method.

### 4.1. Event Camera VS Frame Camera

To show the advantages of event cameras in object tracking, we designed a comparison test in which we fixed a picture on the fan blade, then turned on the fan, let the fan rotate at high speed, and used a frame camera and an event camera to shoot the picture. We visualized the event data for the convenience of demonstration. Figure 5a shows the image when the fan is stationary and Figure 5b shows the image taken with a frame camera when the fan rotates at a high speed, though the picture can no longer be observed due to severe motion blur. Figure 5c shows the image taken with an event camera when the fan rotates at high speed. The event camera avoids motion blur, and a clear target structure without background is captured by the event camera [60].

Our experiments demonstrate that event cameras have a great advantage in intense motion tracking scenarios. Perhaps some ultra-high-speed frame rate cameras can also capture fast-moving targets well; however, their data bandwidth and power consumption are surprisingly large. In the meantime, this example shows one of the advantages of event cameras to capture fast-moving objects with less data redundancy.

### 4.2. Comparison to State-of-the-Art

In order to better evaluate our proposed methods, we compare our proposed approach with state-of-the-art event-based trackers [19,20] and frame-based trackers [42,43,44] on the same dataset [61,62]. This dataset proposes a series of challenging scenarios for high-speed robotics, under three motion settings: translation, rotation, and 6-DoF. This dataset is very challenging for tracking algorithms; it contains rapid movement of the camera with multiple degrees of freedom, scale changes, out-of-view, and occlusion.

**Figure 7:**
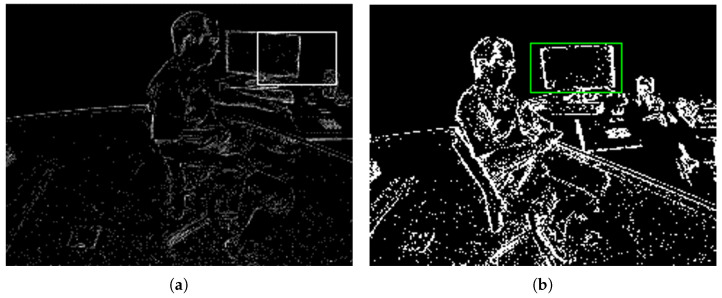

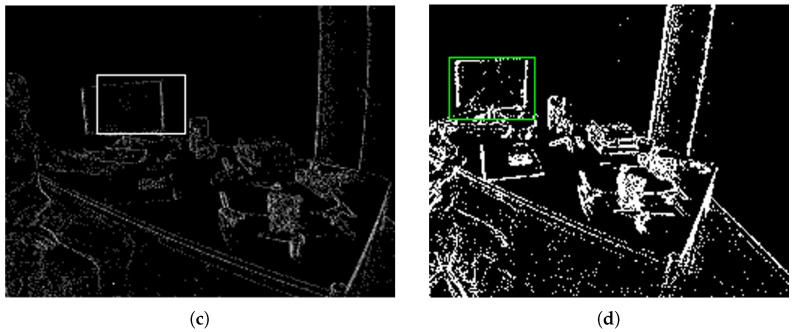
We compared our proposed EVtracker and e-TLD on challenging event data. (**a**,**c**) show the visualization results of e-TLD; (**b**,**d**) show the visualization results of our EVtracker. Our method can balance event resolution and spatial resolution of event data well, and the tracking results of our method are more stable and robust than the results of e-TLD. The white and green boxes represent the bounding box of the tracking.

Our proposed approach obtains better performance compared to e-LOT [20] and e-TLD [19]. As shown in Table 2, our proposed method improves the accuracy of e-TLD by 31.85%; more results are detailed in Table 3. In Figure 7, we visualized our results and compared them with the e-TLD algorithm; we can observe that the event image reconstructed by our method is clearer with higher-quality features.

As shown in Table 4, we compare our EVtracker with state-of-the-art (SOTA) frame-based trackers [42,43,44] on the same dataset. For SOTA frame-based trackers, we use their official long-term tracking model for testing. Since frame-based trackers cannot directly handle asynchronous event streams, we need to adapt them. In this experiment, we only adjusted the Siam family tracking algorithm for comparison. Specifically, we reconstructed asynchronous event streams with fixed parameters(*N* = 30,000) and retrained their backbone. Since the test scenarios are very challenging and traditional methods lack adaptability, conventional frame-based trackers cannot achieve good results.

### 4.3. Ablation Study

To evaluate the effectiveness of our event frame reconstruction strategy for our tracking algorithm, firstly, we analyzed different event image reconstruction strategies through visual comparison. Then, we set the optimal hand-crafted parameters—▽T and *N*—for comparison in ablation experiments. Finally, we evaluated the specific objects of our algorithm in different scenarios, as shown in Table 5. Based on the evaluation of these details, we give our analysis and observations.

**Figure 8:**
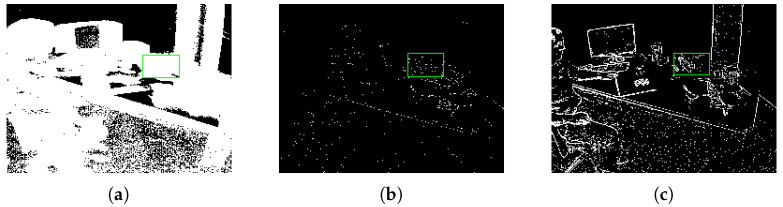
Here we use three strategies to reconstruct the event image. (**a**) shows the event frame reconstructed using the strategy of [28], and (**b**) shows the event image reconstructed using the strategy of [29]. (**c**) shows the event images reconstructed using our proposed strategy. In terms of visual comparison, the accumulation of too many event pixels in event image (**a**) causes the object to be blurred, while the event image (**b**) is too sparse to observe the object. Our event image has a clear object structure and edges. The green box represents the tracking object initialized in the first frame.

As shown in Figure 8a,b, the event image reconstruction method has been successfully applied for other tasks [28,29]. We can easily observe that the event image is very blurry due to the aggregation of too many event pixels in the two-dimensional space in Figure 8a. Unlike in Figure 8a, the event pixels are too few to form a complete event image in Figure 8b. Both Figure 8a,b are failures in the visual task because the event image cannot provide a clear two-dimensional description. Effective event image reconstruction methods can solve the contradiction between high temporal resolution and sparse space in event-based vision. Here, we demonstrate the defect of using a fixed ▽T time-window and fixed *N* number of events; although we can use experience to optimize them artificially, the sensitivity of the parameters in different scenarios still exists. Our ablation experiments will prove that the fixed parameters cannot reach the global optimum.

**Figure 9:**
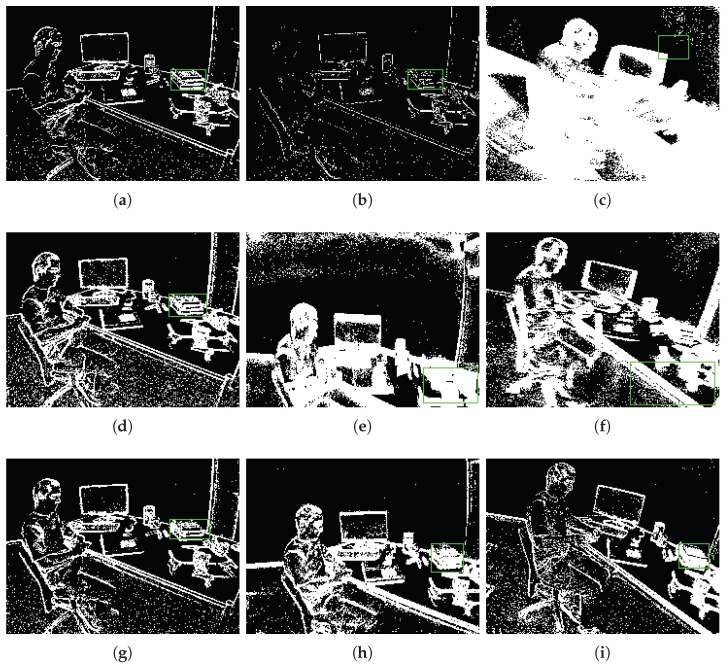
Here, we use three sets of visualized result images for analysis and comparison. (**a**–**c**) show the visualization results of using time windows to gather event data. (**d**–**f**) show the visualization results of gathering event data under the condition of a fixed number of event pixels. (**g**–**i**) show the visualization results of using our dynamic adaptive strategy to gather event data and object tracking. The green box represents the bounding box of the tracking.

We tried many different hand-crafted parameters and selected the two best-performing hand-crafted parameters (*N* = 30,000 and ▽T=30m) for comparison in our ablation experiments. As shown in Table 6 and Table 7, we proposed adaptive strategies to improve performance by 11.28%|7.02% and 13.45%|9.8%, respectively. Despite continuous hand-crafted adjustment and testing, their performance still cannot achieve the global optimal. As shown in Figure 9, we try to gather event data with a fixed time window; the visualization results are shown in (a), (b), (c). Then, we try to gather event data with a fixed number of event pixels; the visualization results are shown in (d) and (e) and (f), (g), (h), (i). These three pictures show the visualization results of using our dynamic adaptive strategy to gather event data and object tracking. From Figure 9, we can observe that the three images (a), (d), (g) all show a good initial state. However, due to the movement of the object or camera in the task scene, the hand-crafted parameters cannot be adjusted adaptively, as shown in Figure 9b,c,e,f, where (b) is too sparse due to low speed and (c), (e), (f) are too dense due to high speed. Our proposed approach provided stable and good output, as show in Figure 9g,h,i. The ablation study proved that our framework can provide an effective event camera reconstruction strategy and object tracking approach. Our dynamic adaptive reconstruction strategy can provide a high-quality event image for event-based vision, and our tracking algorithm is robust and effective.

As shown in Table 5, we evaluated in detail the performance of our algorithm in tracking different objects. We observed that the highest challenge scenario is 6-DoF in all challenge scenarios, because it contains a rotation of six degrees of freedom. Among all tracked objects, we found that the more complex features are more challenging, such as drones. This is due to the lack of robustness of the event space feature. Compared with traditional computer vision methods, the event data lacks large-scale pre-trained models, and the event camera sensor resolution still needs improvement. Therefore, we can improve our algorithm in the future by increasing the spatial resolution of the event camera and using a robust pre-trained model.

### 4.4. Discussion

In this part, we provide discussions based on our experiments and analysis, and mentions the limitations of our method. There are two main reasons why our approach outperforms e-LOT and e-TLD: (1) our method uses more powerful deep learned features, while those methods only use hand-crafted features; (2) our event image reconstruction strategy is more effective than theirs, as shown in Figure 7, so our proposed approach performs better. Our experiments have demonstrated that our method can dynamically adjust the two-dimensional description of event images according to different scenes, and solve challenges in tracking tasks such as motion blur, fast motion, illumination variation, rotation, etc. However, our method still has some limitations. Due to the lack of large-scale event datasets, our network was only trained on small datasets; the features are therefore not robust enough to adapt to all scenarios. Our current convolution networks are designed for synchronous frames and cannot fully utilize all the advantages of event data. Designing methods via few-shot learning and spiking neural networks may be promising approaches to overcome these limitations.

## 5. Conclusions

In this paper, we propose a novel long-term object tracker for event cameras. We also demonstrate the numerous advantages of employing event cameras for object tracking by testing the system with several challenging datasets. To highlight the difference in operating principles between event cameras and conventional cameras, we designed interesting experiments to demonstrate the advantages of event cameras. Additionally, we put forward a strategy for event image reconstruction using feature evaluation. This approach effectively and dynamically balances event data in the spatiotemporal domain and provides adequate two-dimensional spatial information for tracking tasks. We optimized and improved the Siamese network for long-term event-based tracking. Objects are repositioned after they re-enter the field of view of the camera during long-term tracking. Findings from detailed experiments show that our proposed approach yields better accuracy and robustness than other similar methods. For tracking tasks, most current difficulties are caused by the inability to balance the temporal and spatial resolution of images. For instance, low temporal resolution leads to motion blur, uneven temporal and spatial light resolution results in illumination issues, and long conventional camera exposure time causes pixel stacking. Due to the limitations of sensor hardware, most object tracking algorithms focus on extracting high-quality spatial features, but ignore temporal resolution. Based on the high temporal resolution and dynamic capability of event cameras, our method accomplishes challenging tracking tasks that cannot be performed by traditional cameras in several crucial areas such as aerospace, autonomous driving, and drones. We believe that our work will help to solve the inherent challenges that exist in conventional computer vision research and unlock the application potential of event cameras. In future studies, we will extend our target reconstruction algorithm to other areas of event vision, including classification, segmentation, and detection.

## Figures and Tables

**Table 1:** The difference between event cameras and frame cameras.

Features	Event Camera	Frame Camera
Temporal domain	Dense	Sparse
Spatial domain	Sparse	Dense
Redundant data	Low	High
Dynamic range	High	Low
Update model	Asynchronous	Synchronous
Shutter	No	Yes
Power consumption	Low	High

**Table 2:** Comparison to recent SOTA algorithms using the Success Plot metric, and the best results are highlighted bold-faced.

Methods	6-DoF	Translation	Rotation	Avg.
e-LOT [20]	14.17	30.63	27.54	24.11
e-TLD [19]	39.27	70.10	43.80	51.05
EVtracker	**75.88**	**90.24**	**82.59**	**82.90**

**Table 3:** Comparison to recent SOTA algorithm e-LOT [20] and e-TLD [19] using the Success Plot Metric, and the best results are highlighted bold-faced.

6-DoF	Head	Monitor	Drone	Cup	Books	Avg.
e-LOT [20]	6.12	23.84	2.44	31.56	6.92	14.17
e-TLD [19]	31.72	61.74	51.10	22.47	29.33	39.27
EVtracker	**63.44**	**89.09**	**61.29**	**79.38**	**86.22**	**75.88**
**Translation**	**Head**	**Monitor**	**Drone**	**Cup**	**Books**	**Avg.**
e-LOT [20]	14.50	17.69	20.21	74.00	26.47	30.63
e-TLD [19]	58.95	76.43	87.31	59.72	68.09	70.10
EVtracker	**91.21**	**87.27**	**90.97**	**90.72**	**91.04**	**90.24**
**Rotation**	**Head**	**Monitor**	**Drone**	**Cup**	**Books**	**Avg.**
e-LOT [20]	13.49	15.03	24.45	43.59	41.15	27.54
e-TLD [19]	18.32	48.78	53.31	47.77	50.83	43.80
EVtracker	**85.07**	**82.04**	**74.02**	**85.35**	**86.48**	**82.59**

**Table 4:** Comparison to recent SOTA algorithms using the Success Plot metric and Precision Plot metric, and the best results are highlighted bold-faced.

Methods	Success Plot	Precision Plot
SiamRPN [42]	20.91	25.89
SiamRPN++[44]	31.34	36.48
SiamMask [43]	32.74	42.24
EVtracker	**82.90**	**89.38**

**Table 5:** Quantitative tracking results (%) of EVtracker.

6-DoF	Head	Monitor	Drone	Cup	Books
Success Plot	63.44	89.09	61.29	79.38	86.22
Precision Plot	93.70	90.13	60.84	89.06	90.70
**Translation**	**Head**	**Monitor**	**Drone**	**Cup**	**Books**
Success Plot	91.21	87.27	90.97	90.72	91.04
Precision Plot	98.81	86.02	96.40	98.22	98.83
**Rotation**	**Head**	**Monitor**	**Drone**	**Cup**	**Books**
Success Plot	85.07	82.04	74.02	85.35	86.48
Precision Plot	94.23	83.37	78.27	95.06	87.10
**ALL**	**Head**	**Monitor**	**Drone**	**Cup**	**Books**
Success Plot	79.91	86.13	75.43	85.15	87.91
Precision Plot	95.58	86.51	78.50	94.11	92.21

**Table 6:** Using the Success Plot metric for comparison in an ablation study, and the best results are highlighted bold-faced.

Challenges	N	▽T	Adaptivity
6-DoF	69.49	70.76	75.88
Rotation	60.71	87.51	90.24
Translation	84.64	50.10	82.59
Avg.	71.62	69.45	**82.90**

**Table 7:** Using the Precision Plot metric for comparison in an ablation study, and the best results are highlighted bold-faced.

Challenges	N	▽T	Adaptivity
6-DoF	79.05	82.21	84.88
Rotation	72.16	62.29	87.61
Translation	95.86	94.24	95.66
Avg.	82.36	79.58	**89.38**

## Data Availability

Some or all data, models or code generated or used during the study are available from the first author and the corresponding author by request.

## References

[1] Mitrokhin A., Fermüller C., Parameshwara C., Aloimonos Y. Event-based moving object detection and tracking. Proceedings of the 2018 IEEE/RSJ International Conference on Intelligent Robots and Systems (IROS), IEEE.

[2] Chen G., Cao H., Conradt J., Tang H., Rohrbein F., Knoll A. (2020). Event-based neuromorphic vision for autonomous driving: A paradigm shift for bio-inspired visual sensing and perception. IEEE Signal Process. Mag..

[3] Gehrig M., Shrestha S.B., Mouritzen D., Scaramuzza D. Event-based angular velocity regression with spiking networks. Proceedings of the 2020 IEEE International Conference on Robotics and Automation (ICRA), IEEE.

[4] Deng Y., Chen H., Li Y. (2021). MVF-Net: A Multi-view Fusion Network for Event-based Object Classification. IEEE Transactions on Circuits and Systems for Video Technology.

[5] Gallego G., Delbruck T., Orchard G.M., Bartolozzi C., Taba B., Censi A., Leutenegger S., Davison A., Conradt J., Daniilidis K. (2020). Event-based Vision: A Survey. IEEE Transactions on Pattern Analysis and Machine Intelligence.

[6] Liu S.C., Rueckauer B., Ceolini E., Huber A., Delbruck T. (2019). Event-driven sensing for efficient perception: Vision and audition algorithms. IEEE Signal Process. Mag..

[7] Maqueda A.I., Loquercio A., Gallego G., García N., Scaramuzza D. Event-based vision meets deep learning on steering prediction for self-driving cars. Proceedings of the IEEE Conference on Computer Vision and Pattern Recognition (CVPR).

[8] Kim H., Leutenegger S., Davison A.J. Real-time 3D reconstruction and 6-DoF tracking with an event camera. Proceedings of the European Conference on Computer Vision (ECCV).

[9] Vitale A., Renner A., Nauer C., Scaramuzza D., Sandamirskaya Y. Event-driven vision and control for UAVs on a neuromorphic chip. Proceedings of the 2021 IEEE International Conference on Robotics and Automation (ICRA).

[10] He W., Wu Y., Deng L., Li G., Wang H., Tian Y., Ding W., Wang W., Xie Y. (2020). Comparing snns and rnns on neuromorphic vision datasets: Similarities and differences. Neural Netw..

[11] Chen X., Yan B., Zhu J., Wang D., Yang X., Lu H. Transformer tracking. Proceedings of the IEEE/CVF Conference on Computer Vision and Pattern Recognition (CVPR).

[12] Yin T., Zhou X., Krahenbuhl P. Center-based 3d object detection and tracking. Proceedings of the IEEE/CVF Conference on Computer Vision and Pattern Recognition (CVPR).

[13] Marvasti-Zadeh S.M., Cheng L., Ghanei-Yakhdan H., Kasaei S. (2021). Deep learning for visual tracking: A comprehensive survey. IEEE Transactions on Intelligent Transportation Systems.

[14] Zhang Y., Wang L., Qi J., Wang D., Feng M., Lu H. Structured siamese network for real-time visual tracking. Proceedings of the European conference on computer vision (ECCV).

[15] Wu Y., Lim J., Yang M.H. Online object tracking: A benchmark. Proceedings of the IEEE Conference on Computer Vision and Pattern Recognition (CVPR).

[16] Kristan M., Leonardis A., Matas J., Felsberg M., Pflugfelder R., Kämäräinen J.K., Danelljan M., Zajc L.Č., Lukežič A., Drbohlav O. The eighth visual object tracking VOT2020 challenge results. Proceedings of the European Conference on Computer Vision (ECCV).

[17] Mueggler E., Huber B., Scaramuzza D. Event-based, 6-DOF pose tracking for high-speed maneuvers. Proceedings of the 2014 IEEE/RSJ International Conference on Intelligent Robots and Systems (IROS).

[18] Seifozzakerini S., Yau W.Y., Zhao B., Mao K. Event-Based Hough Transform in a Spiking Neural Network for Multiple Line Detection and Tracking Using a Dynamic Vision Sensor. Proceedings of the British Machine Vision Conference (BMVC).

[19] Ramesh B., Zhang S., Yang H., Ussa A., Ong M., Orchard G., Xiang C. (2021). e-TLD: Event-Based Framework for Dynamic Object Tracking. IEEE Trans. Circuits Syst. Video Technol..

[20] Ramesh B., Yang H., Orchard G., Le Thi N.A., Zhang S., Xiang C. (2019). Dart: Distribution aware retinal transform for event-based cameras. IEEE Trans. Pattern Anal. Mach. Intell..

[21] Pan L., Scheerlinck C., Yu X., Hartley R., Liu M., Dai Y. Bringing a blurry frame alive at high frame-rate with an event camera. Proceedings of the IEEE/CVF Conference on Computer Vision and Pattern Recognition (CVPR).

[22] Huang J., Wang S., Guo M., Chen S. (2018). Event-guided structured output tracking of fast-moving objects using a celex sensor. IEEE Trans. Circuits Syst. Video Technol..

[23] Duo J., Zhao L. (2021). An Asynchronous Real-Time Corner Extraction and Tracking Algorithm for Event Camera. Sensors.

[24] Furmonas J., Liobe J., Barzdenas V. (2022). Analytical review of event-based camera depth estimation methods and systems. Sensors.

[25] Ozawa T., Sekikawa Y., Saito H. (2022). Accuracy and Speed Improvement of Event Camera Motion Estimation Using a Bird’s-Eye View Transformation. Sensors.

[26] Orchard G., Meyer C., Etienne-Cummings R., Posch C., Thakor N., Benosman R. (2015). HFirst: A temporal approach to object recognition. IEEE Trans. Pattern Anal. Mach. Intell..

[27] Lee J.H., Delbruck T., Pfeiffer M., Park P.K., Shin C.W., Ryu H., Kang B.C. (2014). Real-time gesture interface based on event-driven processing from stereo silicon retinas. IEEE Trans. Neural Netw. Learn. Syst..

[28] Vidal A.R., Rebecq H., Horstschaefer T., Scaramuzza D. (2018). Ultimate SLAM? Combining events, images, and IMU for robust visual SLAM in HDR and high-speed scenarios. IEEE Robot. Autom. Lett..

[29] Xu L., Xu W., Golyanik V., Habermann M., Fang L., Theobalt C. Eventcap: Monocular 3d capture of high-speed human motions using an event camera. Proceedings of the IEEE/CVF Conference on Computer Vision and Pattern Recognition (CVPR).

[30] Bagchi S., Chin T.J. Event-based star tracking via multiresolution progressive Hough transforms. Proceedings of the IEEE/CVF Winter Conference on Applications of Computer Vision(WACV).

[31] Mei X., Ling H., Wu Y., Blasch E.P., Bai L. (2013). Efficient minimum error bounded particle resampling L1 tracker with occlusion detection. IEEE Trans. Image Process..

[32] Bao C., Wu Y., Ling H., Ji H. Real time robust l1 tracker using accelerated proximal gradient approach. Proceedings of the 2012 IEEE Conference on Computer Vision and Pattern Recognition (CVPR).

[33] Li H., Shen C., Shi Q. Real-time visual tracking using compressive sensing. Proceedings of the 2011 IEEE Conference on Computer Vision and Pattern Recognition (CVPR).

[34] Zhang K., Zhang L., Yang M.H. (2014). Fast compressive tracking. IEEE Trans. Pattern Anal. Mach. Intell..

[35] Kalal Z., Mikolajczyk K., Matas J. (2011). Tracking-learning-detection. IEEE Trans. Pattern Anal. Mach. Intell..

[36] Wang S., Lu H., Yang F., Yang M.H. Superpixel tracking. Proceedings of the 2011 International Conference on Computer Vision (ICCV).

[37] Yang F., Lu H., Yang M.H. (2014). Robust superpixel tracking. IEEE Trans. Image Process..

[38] Yuan Y., Fang J., Wang Q. (2013). Robust superpixel tracking via depth fusion. IEEE Trans. Circuits Syst. Video Technol..

[39] Yu Y., Xiong Y., Huang W., Scott M.R. Deformable siamese attention networks for visual object tracking. Proceedings of the IEEE/CVF Conference on Computer Vision and Pattern Recognition (CVPR).

[40] Ciaparrone G., Sánchez F.L., Tabik S., Troiano L., Tagliaferri R., Herrera F. (2020). Deep learning in video multi-object tracking: A survey. Neurocomputing.

[41] Cen M., Jung C. Fully convolutional siamese fusion networks for object tracking. Proceedings of the 2018 25th IEEE International Conference on Image Processing (ICIP), IEEE.

[42] Li B., Yan J., Wu W., Zhu Z., Hu X. High performance visual tracking with siamese region proposal network. Proceedings of the IEEE conference on computer vision and pattern recognition (CVPR).

[43] Wang Q., Zhang L., Bertinetto L., Hu W., Torr P.H. Fast online object tracking and segmentation: A unifying approach. Proceedings of the IEEE/CVF Conference on Computer Vision and Pattern Recognition.

[44] Li B., Wu W., Wang Q., Zhang F., Xing J., Yan J. Siamrpn++: Evolution of siamese visual tracking with very deep networks. Proceedings of the IEEE/CVF Conference on Computer Vision and Pattern Recognition (CVPR).

[45] Afshar S., Nicholson A.P., van Schaik A., Cohen G. (2019). Event-based object detection and tracking for space situational awareness. arXiv.

[46] Chin T.J., Bagchi S., Eriksson A., Van Schaik A. Star tracking using an event camera. Proceedings of the IEEE/CVF Conference on Computer Vision and Pattern Recognition Workshops (CVPRW).

[47] Hinz G., Chen G., Aafaque M., Röhrbein F., Conradt J., Bing Z., Qu Z., Stechele W., Knoll A. (2017). Online multi-object tracking-by-clustering for intelligent transportation system with neuromorphic vision sensor. Proceedings of the Joint German/Austrian Conference on Artificial Intelligence (Künstliche Intelligenz).

[48] Chen G., Cao H., Aafaque M., Chen J., Ye C., Röhrbein F., Conradt J., Chen K., Bing Z., Liu X. (2018). Neuromorphic vision based multivehicle detection and tracking for intelligent transportation system. J. Adv. Transp..

[49] Chen G., Wang F., Li W., Hong L., Conradt J., Chen J., Zhang Z., Lu Y., Knoll A. (2020). NeuroIV: Neuromorphic vision meets intelligent vehicle towards safe driving with a new database and baseline evaluations. IEEE Trans. Intell. Transp. Syst..

[50] Chen G., Hong L., Dong J., Liu P., Conradt J., Knoll A. (2020). EDDD: Event-based drowsiness driving detection through facial motion analysis with neuromorphic vision sensor. IEEE Sens. J..

[51] Cao Z., Cheng L., Zhou C., Gu N., Wang X., Tan M. (2015). Spiking neural network-based target tracking control for autonomous mobile robots. Neural Comput. Appl..

[52] Li H., Shi L. (2019). Robust event-based object tracking combining correlation filter and CNN representation. Front. Neurorobotics..

[53] Liu H., Moeys D.P., Das G., Neil D., Liu S.C., Delbrück T. Combined frame-and event-based detection and tracking. Proceedings of the 2016 IEEE International Symposium on Circuits and systems (ISCAS).

[54] Wang X., Li J., Zhu L., Zhang Z., Chen Z., Li X., Wang Y., Tian Y., Wu F. (2021). VisEvent: Reliable Object Tracking via Collaboration of Frame and Event Flows. arXiv.

[55] Calabrese E., Taverni G., Awai Easthope C., Skriabine S., Corradi F., Longinotti L., Eng K., Delbruck T. Dhp19: Dynamic vision sensor 3d human pose dataset. Proceedings of the IEEE/CVF Conference on Computer Vision and Pattern Recognition Workshops (CVPRW).

[56] Bochkovskiy A., Wang C.Y., Liao H.Y.M. (2020). Yolov4: Optimal speed and accuracy of object detection. arXiv.

[57] Voigtlaender P., Luiten J., Torr P.H., Leibe B. Siam r-cnn: Visual tracking by re-detection. Proceedings of the IEEE/CVF Conference on Computer Vision and Pattern Recognition (CVPR).

[58] Ondrašovič M., Tarábek P. (2021). Siamese Visual Object Tracking: A Survey. IEEE Access.

[59] Krizhevsky A., Sutskever I., Hinton G.E. (2012). Imagenet classification with deep convolutional neural networks. Adv. Neural Inf. Process. Syst..

[60] Chen S., Guo M. Live demonstration: CeleX-V: A 1M pixel multi-mode event-based sensor. Proceedings of the 2019 IEEE/CVF Conference on Computer Vision and Pattern Recognition Workshops (CVPRW).

[61] Kueng B., Mueggler E., Gallego G., Scaramuzza D. Low-latency visual odometry using event-based feature tracks. Proceedings of the 2016 IEEE/RSJ International Conference on Intelligent Robots and Systems (IROS).

[62] Gallego G., Scaramuzza D. (2017). Accurate angular velocity estimation with an event camera. IEEE Robot. Autom. Lett..

